# Precision targeting of food biofilm-forming genes by microbial scissors: CRISPR-Cas as an effective modulator

**DOI:** 10.3389/fmicb.2022.964848

**Published:** 2022-08-09

**Authors:** Sreejita Ghosh, Dibyajit Lahiri, Moupriya Nag, Tanmay Sarkar, Siddhartha Pati, Hisham Atan Edinur, Manoj Kumar, Muhammad R. A. Mohd Zain, Rina Rani Ray

**Affiliations:** ^1^Department of Biotechnology, Maulana Abul Kalam Azad University of Technology, Kolkata, India; ^2^Department of Biotechnology, University of Engineering and Management, Kolkata, India; ^3^Department of Food Processing Technology, Malda Polytechnic, West Bengal State Council of Technical Education, Govt. of West Bengal, Malda, India; ^4^Skills Innovation and Academic Network (SIAN) Institute, Association for Biodiversity Conservation and Research (ABC), Balasore, India; ^5^NatNov Bioscience Private Limited, Balasore, India; ^6^School of Health Sciences, Health Campus, Universiti Sains Malaysia, Kubang Kerian, Kelantan, Malaysia; ^7^Chemical and Biochemical Processing Division, ICAR-Central Institute for Research on Cotton Technology, Mumbai, Maharashtra, India; ^8^Department of Orthopaedics, School of Medical Sciences, Universiti Sains Malaysia, Kubang Kerian, Kelantan, Malaysia

**Keywords:** AMR, CRISPRi, food microbiology, biofilm, gene editing, virulence, food safety

## Abstract

The abrupt emergence of antimicrobial resistant (AMR) bacterial strains has been recognized as one of the biggest public health threats affecting the human race and food processing industries. One of the causes for the emergence of AMR is the ability of the microorganisms to form biofilm as a defense strategy that restricts the penetration of antimicrobial agents into bacterial cells. About 80% of human diseases are caused by biofilm-associated sessile microbes. Bacterial biofilm formation involves a cascade of genes that are regulated *via* the mechanism of quorum sensing (QS) and signaling pathways that control the production of the extracellular polymeric matrix (EPS), responsible for the three-dimensional architecture of the biofilm. Another defense strategy utilized commonly by various bacteria includes clustered regularly interspaced short palindromic repeats interference (CRISPRi) system that prevents the bacterial cell from viral invasion. Since multigenic signaling pathways and controlling systems are involved in each and every step of biofilm formation, the CRISPRi system can be adopted as an effective strategy to target the genomic system involved in biofilm formation. Overall, this technology enables site-specific integration of genes into the host enabling the development of paratransgenic control strategies to interfere with pathogenic bacterial strains. CRISPR-RNA-guided Cas9 endonuclease, being a promising genome editing tool, can be effectively programmed to re-sensitize the bacteria by targeting AMR-encoding plasmid genes involved in biofilm formation and virulence to revert bacterial resistance to antibiotics. CRISPRi-facilitated silencing of genes encoding regulatory proteins associated with biofilm production is considered by researchers as a dependable approach for editing gene networks in various biofilm-forming bacteria either by inactivating biofilm-forming genes or by integrating genes corresponding to antibiotic resistance or fluorescent markers into the host genome for better analysis of its functions both *in vitro* and *in vivo* or by editing genes to stop the secretion of toxins as harmful metabolites in food industries, thereby upgrading the human health status.

## Introduction

The ever-increasing occurrence of bacterial biofilms in foods led to the emergence of a wide variety of outcomes, starting from negative outcomes like spoilage of food products and foodborne diseases to positive outcomes including preservation of food and improved health of the gut (Ghosh et al., 2021; Kirtonia et al., 2021). Naturally, many matrices of food are composed of suitable conditions for the growth of bacteria due to the existence of important substrates, such as lipids, carbohydrates, minerals, proteins, and vitamins (Papadimitriou et al., 2015). A surplus of nutrients in addition to the presence of solid substrata will lead to the proliferation of microbial cells resulting in biofilm formation that is impenetrable to a wide variety of antimicrobials. This has resulted in the emergence of antimicrobial resistant strains that are difficult to remove (Lebeaux et al., 2014).

The discovery of antibiotics is one of the biggest milestones achieved in medicines, which led to the prevention of a wide variety of diseases in humans. In contrast to this, misuse and overuse of antibiotics have led to the development of antibiotic resistance in bacteria (Ventola, 2015). Multi-drug resistant (MDR) bacteria are the major challenges that are being faced by the fields of medicine and food microbiology (Prestinaci et al., 2015). In 2017, World Health Organization (WHO) proposed a list of bacteria as some of the most dangerous and pathogenic, which includes *Enterococcus faecium, Staphylococcus aureus, Klebsiella pneumoniae, Acinetobacter baumanii, Pseudomonas aeruginosa*, and *Enterobacter* spp. (ESKAPE), and all these bacteria are responsible for hospital-acquired infections and some are also involved in causing food spoilage (*S. aureus*) (Asokan et al., 2019). This group of bacteria is known as superbugs because the available antibiotics are not sufficient to eradicate the infections caused by them. These superbugs contain resistant genes within their plasmids, thereby spreading the development of MDR at an alarming rate (Nikaido, 2009). Therefore, it is of the utmost need to develop some novel strategies to combat these bacteria by certain genome editing techniques, which will alter the resistant genes and make them more susceptible to antibiotic therapies or by inhibiting the genes involved in QS and thus hindering the formation of biofilm (Wan et al., 2021).

Over recent years, advancements in genomics and molecular biology have enhanced the ability of the food industries to manage and monitor the unfavorable food microbiota (Arnold et al., 2016). Amongst these recent advancements, one of them is the technology of CRISPR-Cas 9 (CRISPR-associated sequences). When external genetic elements (like bacteriophages) invade the bacterial cells, an immune response is mounted due to the presence of a defense mechanism, which is described as the CRISPR-Cas system (Marchfelder, 2013; Chen and Chu, 2019; Mitsui et al., 2019). CRISPR and the different genes associated with it possess the capacity of providing a promising answer to emerging resistance against antibiotics (Gholizadeh et al., 2020). A typical system of CRISPR-Cas is composed of three constituents: (a) an operon having a Cas gene group, (b) a leader sequence, and (c) an array of CRISPR DNA (Barrangou et al., 2007). The CRISPR-Cas technology has developed very rapidly as a tool for genome editing in different setups of experiments and types of cells. Results from experimental data have indicated its application in targeting antibacterial resistance genes (ARGs) possessing high rates of specificity and sensitivity (Uddin et al., 2020).

The biology of CRISPR is embedded in the microbiology of food, which is confirmed by its function in adaptive immunity against *Streptococcus thermophilus* phages (the main starter culture for yogurt manufacturing) (Barrangou et al., 2007). In the last decade, CRISPR research has exponentially grown mainly because of its capability to execute specific cleavage of DNA, so that it can be used for genome editing. With the emergence of terms like “CRISPR revolution” and “CRISPR craze,” and the pace at which the scientific world has characterized and understood native systems of CRISPR-Cas, it is evident that this technology will be used in the form of genetic tools for gene insertion, deletion, and regulation in non-native systems (Rodolphe, 2014). The utilization of CRISPR-associated technologies in prokaryotes that are used in the food industries is also vast.

## Biology of CRISPR-Cas

The repeating spacer arrays of CRISPR in combination with Cas proteins form the RNA-mediated and DNA-encoded adaptive immunity in archaea and bacteria, thus protecting against the phages and other invasive mobile genetic elements (MGEs) through RNA or DNA cleavage (Barrangou et al., 2007). The array of repeat-spacer comprises a leader sequence that can serve as a promoter for transcription, subsequently leading to a conserved palindromic repeat sequence flanking the unique spacers. This array of repeat-spacer sequences was first identified in *Escherichia coli* strain K12 in the year of 1987, although the word “CRISPR” was not coined till then to describe them (Ishino et al., 1987). The origin and purpose of the arrays of repeat-spacers were not known till 2005, when three research groups observed resemblances between bacteriophage and spacer sequences and MGEs (Bolotin et al., 2005; Mojica et al., 2005; Pourcel et al., 2005). Within a year, it was projected that CRISPR was responsible for heritable cellular immunity *via* interference on the basis of the composition of amino acids and functions of the domains of the Cas proteins (Makarova et al., 2006). CRISPR provides adaptive phage resistivity, and the Cas genes associated with CRISPR form an integral constituent of both immunity and vaccination (Barrangou et al., 2007). Researchers have indicated that CRISPR helps in the targeting of DNA, thereby preventing the uptake of plasmids and subsequently solidifying the function of CRISPR in cell-mediated immunity against exogenous DNA, and this study sparked immense interest in the RNA-driven mechanisms of CRISPR-Cas (Marraffini and Sontheimer, 2010).

**Figure 1:**
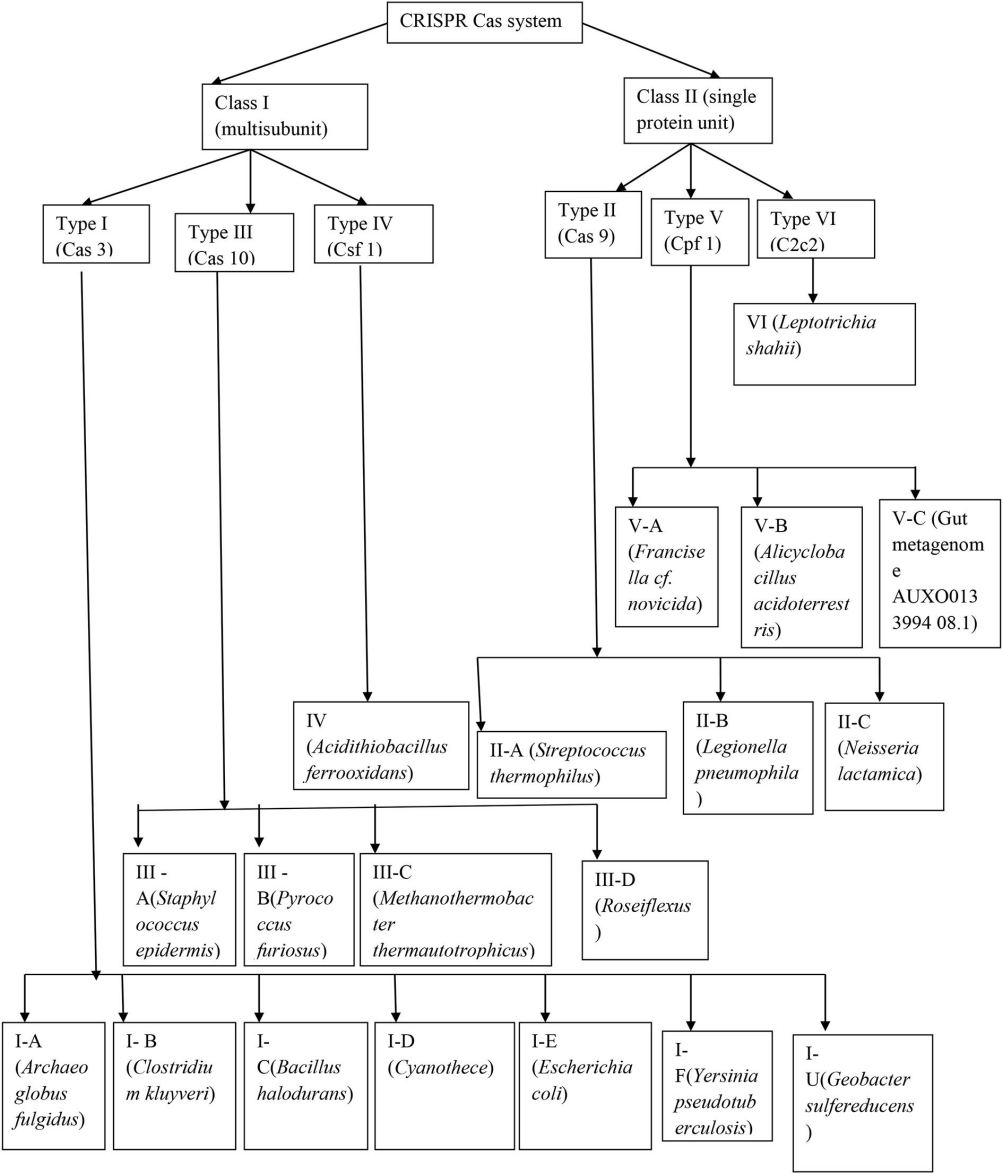
Detailed structure of the CRISPR-Cas system.

The emergence of CRISPR-Cas resulted in the identification of a diverse array of CRISPR-Cas systems, which can be differentiated from each other by the presence of signature genes, the processing mechanism of precursor CRISPR RNA (pre-crRNA), and the process through which the target RNA or DNA is cleaved (Makarova et al., 2015). The most recent system of classification has described six main types of CRISPR-Cas systems (I–VI), which include 19 subtypes that fall under two major categories described by the complex of crRNA-effector, which brings about the RNA or DNA cleavage in the interference step (Figure 1).

### CRISPR-Cas-mediated adaptation

The process of adaption comprises two steps, in which the system of CRISPR-Cas needs a new spacer sequence *via* the sampling of foreign DNA followed by implantation of the novel target sequences within its repeat-spacer array (Sternberg et al., 2014). When the target sequence exists inside the foreign DNA, it is called a protospacer, while after being incorporated within the repeat-spacer array, it is called as a spacer. Adaptation is carried out by the nearly universal complex formed by Cas1-Cas2, and all the six different kinds of systems of CRISPR-Cas consists of the Cas1 and Cas2 genes except for the type IV CRISPR-Cas system (Makarova et al., 2015).

Inside the type I and type II CRISPR-Cas systems, the selection procedure of the spacers is mediated by the protospacer adjacent motif (PAM), which is a distinctive series of 2–4 nucleotides flanking the protospacer, thereby marking it as the target sequence (Mojica et al., 2009). Not only is PAM important for the selection process to occur followed by protospacer binding, but it also assures that a difference is made between the foreign DNA and the host due to the existence of PAM within the target DNA and not inside the CRISPR loci (Marraffini and Sontheimer, 2010). Type V systems even make use of a PAM, which is dependent on the selection of protospacer, even though adaptation has still not been well-characterized in these systems of CRISPR (Zetsche et al., 2015). Type III CRISPR-Cas systems are known to make use of a PAM, which is not dependent on the process of adaptation, even though the exact mechanism is still unclear (van der Oost et al., 2014). Apart from this, biases in the selection of spacers also exist like priming, where the already existing spacers have an impact on the acquirement of extra spacers from the same target (Richter et al., 2014).

**Figure 2:**
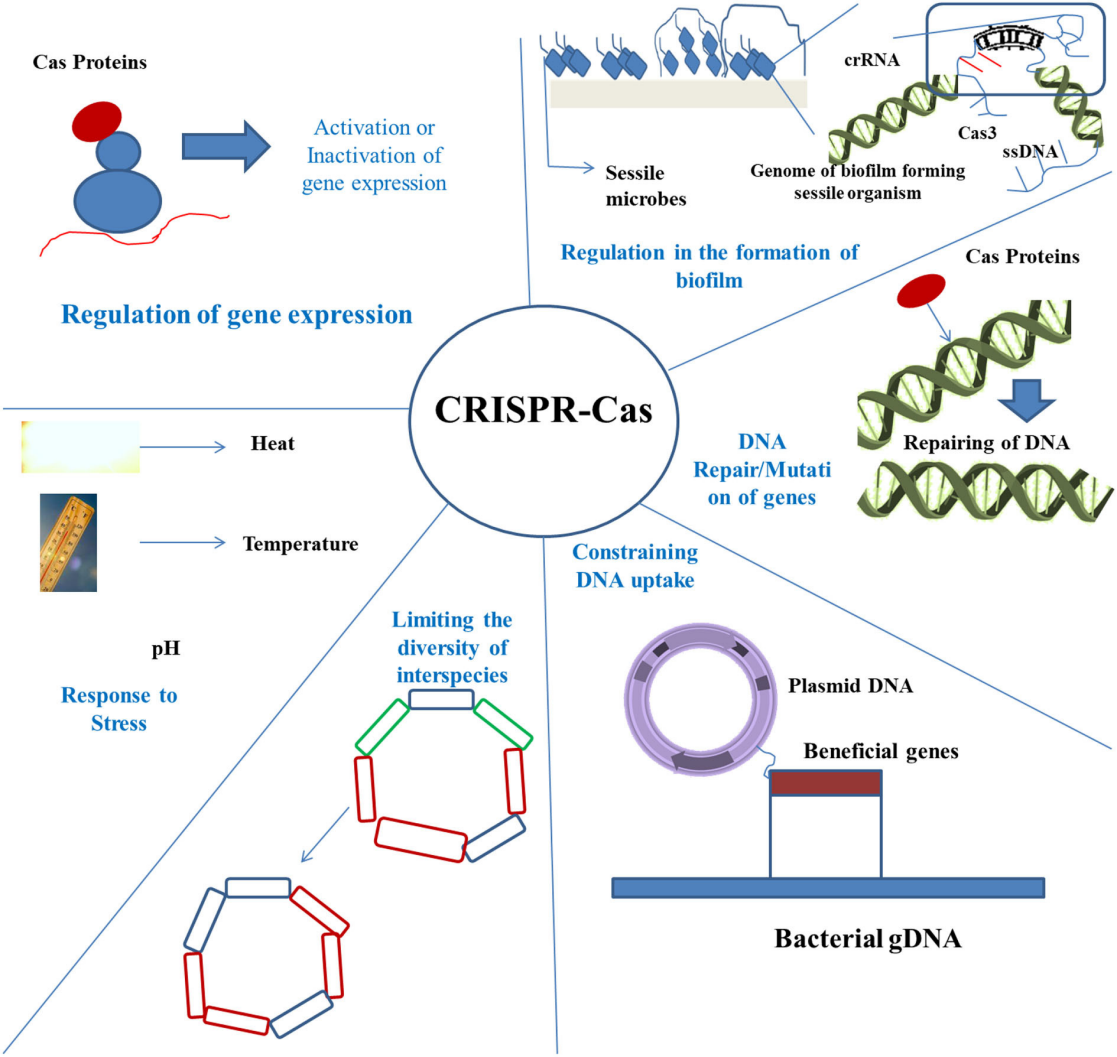
Application of CRISPR-Cas in strategies to control biofilm. There are various mechanisms rendered by the CRISPR-Cas technique, including regulation of the biofilm formation, repair of DNA, preventing the uptake of DNA, bringing about limitation in species diversity, and controlling under various stress conditions.

Once the protospacer is identified through interactions with the PAM, the complex processes the substrate of the foreign DNA into precursors of spacers of a specific size (van der Oost et al., 2014). Cas2 protects the double-stranded DNA in the target sequence inside the complex during this processing. The precursors of the spacer and the complex of Cas1–Cas2 can now localize into the repeat-spacer array, in which Cas1 brings about a staggered cut across the repeat, which is nearest to the leader end and produces two single-stranded repeats (Arslan et al., 2014). The spacer gets integrated followed by ligation between those two repeats, as a result of which the machinery of DNA repair complements these repeats, ending the process of adaptation (Arslan et al., 2014). Since the process of adaptation is chronological and polarized consisting of new spacers, which every time gets incorporated within the proximal-leader end, this repeat-spacer array is considered as historical proof of the events. Since the ingredients of the repeat-spacer array determine the extent to which the bacterial cell is being shielded from the invasions of MGEs, the process of adaptation is important in CRISPR-mediated cell immunity (Figure 2).

### CRISPR-mediated expression process

The process of expression includes multiple steps, which are responsible for crRNA biogenesis. Primarily, the transcription of the repeat-spacer array takes place, which is followed by the transcript processing of the repeat-spacer array, which then gets converted to mature small crRNAs, and ultimately, these mature crRNAs assemble to form the crRNA-effector complex. The leader portion of the CRISPR loci is present directly upstream of the repeat-spacer array and frequently consists of a promoter initiating array transcription (Carte et al., 2014). Usually, the array gets transcribed into a long pre-crRNA, which gets subsequently processed into mature and smaller crRNAs through repeat sequence cleavage. Analyses of these small sequences of RNA have revealed that the profusion of individual crRNAs may change across the loci. Typically, the abundance of crRNA slowly gets reduced from the leader-proximal end to the leader-distal end of the array. In addition to this, ancestral spacers seem more likely to go through deletions from the repeat-spacer array. From an evolutionary and biological point of view, new spacer incorporations at the leader-proximal end having a transcriptional promoter provide advantages to the cell, since novel spacers are more likely to get highly transcribed, thereby facilitating the cell to protect itself effectively against more number of new threats. Once the repeat-spacer array has been transcribed, the processing of pre-crRNA to mature crRNAs undergoes modification because it depends on the type of CRISPR system.

The class I CRISPR system consisting of type I and type III systems are analogous in their processing methods of pre-crRNA. Even though the type IV system theoretically belongs to the class I CRISPR system, expression is yet to be characterized in this system (Makarova et al., 2015). Both type I and type III systems make use of a Cas6-like protein for pre-crRNA processing. Because of the palindromic nature of these repeats, hairpin structures are formed inside these repeat sequences of the pre-crRNAs frequently. Cas6 can hydrolyze the phosphodiester bond present at the 3′ end of each of the repeat hairpin structures forming individual crRNAs. The variants of Cas6 belonging to type I-A, I-B, and I-D systems and also type III systems subsequently transport the crRNAs to their relevant effector complexes (Staals et al., 2013). Variants of Cas6 belonging to the systems type I-E and I-F transport the crRNA to the effector complexes; nevertheless, they even bind to the crRNA strongly and become a major part of the effector complexes. Within the systems of type I-C, a variant of Cas5 is utilized for cleaving the pre-crRNA instead of using Cas6. In contrast to this, the ultimate result is very similar to that of Cas6 because the variant of Cas5 can also become a segment of the effector complex similar to the systems of type I-E and type I-F (Nam et al., 2012). Since the type I and type III systems belong to the multiple subunit-containing class I system of CRISPR, more proteins required to form the complex of crRNA and effector are transported to the complex at this stage.

Class II CRISPR systems, including type VI, type V, and type II systems, differ from class I CRISPR systems based on the pre-crRNA processing method. Type II systems of CRISPR mediate pre-crRNA processing *via* a *trans*-activating crRNA (tracrRNA). This tracrRNA is composed of a sequence, which is complementary to the repeats present within the pre-crRNA. The anti-repeat sequences of the tracrRNA get associated with the pre-crRNA repeats. Cas9 identifies and binds with the complex formed by crRNA-tracrRNA because of the presence of its recognition (REC) lobe (Hirano et al., 2016).

At this stage, RNase III, which is a housekeeping ribonuclease, mediates the processing of the complex and separates it into crRNA and tracrRNA repeat units *via* cleavage of the repeat-anti-repeat segment of each of the crRNA-tracrRNA units. A secondary nuclease (which is still not known) subsequently trims the spacer sequence present at the 5′ end of the crRNA, thus ending this process. The protein Cas9 remains tightly attached to the entirely processed complex formed by pre-crRNA-tracrRNA containing a single protein of the type II crRNA-effector complex. Type VI and type V CRISPR systems were relatively recently discovered; however, their expression mechanism is still uncharacterized. On the other hand, it is known that the type V-A system has the ability to process pre-crRNA by solely using its signature protein Cpf1 (Fonfara et al., 2016). Even though no putative sequences of tracrRNA were found in type V-C systems, putative sequences of tracrRNA have been found in many types of type V-B systems, and hence the cleavage of target DNA is crRNA-specific and dependent on tracrRNA (Shmakov et al., 2015). The maturation of crRNA within type VI systems does not depend on tracrRNA.

### CRISPR-Cas interference

The appropriately formed crRNA-effector complex mediates interference, thereby protecting the cell against the invasion of MGEs through sequence-specific identification and cleaving of the target sequences of nucleic acids. Interference takes place when the spacer sequence present in crRNA interacts with the complementary sequences present within the target protospacers; following the recognition of the protospacer target by the system, endonucleolytic Cas proteins subsequently cleave the invasive target sequence.

Due to the similarities in their crRNA-effector complexes, the class I CRISPR systems (type I and type III) are first discussed followed by the description of class II CRISPR systems (type II, type V, and type VI). However, interference is still uncharacterized in the type IV system of CRISPR.

Interference within type I-E CRISPR system is well-studied and characterized. The crRNA-effector complex along with its multiple subunits is called a CRISPR-associated complex for antiviral defense (Cascade), which goes through the cell searching for MGEs by checking the complementary sequences of DNA (van der Oost et al., 2014). On recognition of the PAM sequence by Cse1, a signal indicating the presence of non-self DNA reaches the crRNA-effector complex followed by destabilization of the DNA duplex and facilitating the crRNA to bind to the target DNA. The spacer sequence present in the crRNA tries to form a base pair with the target sequence, and if the seed sequence of the crRNA mismatches with that of the target sequences, the complex formed by the Cascade gets detached hindering the cleavage of DNA (Zhao et al., 2014). On the other hand, if this results in sufficient base pair formation, an R-loop gets generated, which seems to activate or recruit the signature Cas3 nuclease-helicase to the complex formed by Cascade, which then influences the extensive and progressive degradation of DNA (Huo et al., 2014).

The subtypes of the type III CRISPR system, III-A and III-B, make use of a complex formed by Cmr and Csm proteins, respectively, but still both the complexes utilize the type III signature gene called Cas10. In contrast to type I and type II CRISPR systems, the type III system of CRISPR does not utilize PAMs for distinguishing between non-self and self DNA during the process of interference. Moreover, the type III-A CRISPR system uses a tag on the 5′ crRNA end, which contains eight nucleotides of the repeat sequence. If this tag gets bound to the template DNA strand in addition to the crRNA, the cell can recognize the sequence as self and hence restricts interference. If the csm complex recognizes a target sequence as foreign, it degrades that DNA. The cmr complex present in type III-B CRISPR systems is the first CRISPR-Cas crRNA-effector complex to be found, and it can mediate the cleavage of RNA as well (Hale et al., 2009). The cmr complex recognizes RNA, which is complementary to the sequence of crRNA and seems to use cmr4 for the cleavage of the target RNA at an interval of six nucleotides. Even though it was initially thought that the csm complex of type III-A CRISPR systems targets DNA and the cmr complex of type III-B CRISPR systems targets RNA, recent investigations have unraveled a more complex observation. The csm complex of the type III-A CRISPR systems present in *Thermus thermophilus* and *Sreptococcus thermophilus* recognize and degrade RNA in a manner that is identical to that of the cmr protein complex (Tamulaitis et al., 2014). In addition to this, the cmr complex of the type III-B CRISPR system present in *Sulfolobus islandicus* was found to degrade DNA besides RNA, therefore making it the first CRISPR system to display dual-targeting action (Peng et al., 2015). Apart from this, it seems that in some of the systems, which contain both types of type III CRISPR, like in the case of *T. thermophilus*, the cmr and csm complexes might share crRNA (Staals et al., 2014). There still remains much more to be studied regarding the process of interference in type III CRISPR systems.

The process of interference in the type II CRISPR system is different from that observed in type III and type I systems of CRISPR because of the straightforwardness of its crRNA-effector complex. Apart from the crRNA, the only other remaining constituents are the Cas9 endonuclease and the tracrRNA. Cas9 protein consists of two lobes: the REC lobe helps in the formation of the crRNA-tracrRNA complex by searching the target DNA and the nuclease (NUC) domain mediates the cleavage of the double-stranded target DNA through its RuvC and HNH domains of nuclease (Nishimasu et al., 2015). Type II interference complex screens the DNA *via* indiscrimination in the colliding of the DNA sequences and searching for PAM sequences. When the complex recognizes and gets bound to the PAM on the strand that is non-complementary, the target sequence gets destabilized forming the R-loop. After this, the DNA sequence of the target is scanned to ensure proper complementation with the spacer sequence across the crRNA. If any types of mismatches are found within the seed segment of the protospacer, the complex of crRNA-effector gets detached from the sequence, hindering the event of cleavage (Sternberg et al., 2014). On the other hand, if sufficient complementarity is absent, cleavage of the double-stranded target takes place through the NUC domain or lobe of the protein Cas9 (Giedrius et al., 2012). Contrary to type III and type I systems of CRISPR, which use complexes with multiple subunits for the degradation of the target DNA at several locations, the specificity and simplicity of the interference in native as well as in engineered type II CRISPR make it a unique and useful technique in the field of genome editing, which is driving a huge part of the CRISPR research in today's world.

In the *Francisella* subspecies, type V CRISPR was first identified in 2013, but interference in type V-A and type V-B CRISPR has been characterized over recent years (Yamano et al., 2016). Interference within type V-A CRISPR, recognized by its signature protein Cpf1, has been studied in more detail till now. Type V systems of CRISPR are similar to those of type II CRISPR systems in their utilization of the PAM sequence, using only one protein in their crRNA-effector complexes, and the mechanism by which the cleavage of DNA takes place (Zetsche et al., 2015). When the crRNA-effector complex of type V-A CRISPR recognizes a suitable target by means of PAM, it primarily decides if there is adequate complementarity between the spacer sequence of crRNA and the protospacer. If there are no mismatches within the seed segment, it indicates that a unique RuvC domain and a nuclease domain are responsible for making a staggered cut inside the protospacer having a 5′ overhang at the distal protospacer end from the seed segment and PAM sequence. Systems of type V-A are held as an interesting alternate strategy in comparison to type II CRISPR systems in the field of genome editing. The absence of tracrRNA and the small size of Cpf1 as compared to Cas9 facilitates the manufacture of small effector complexes, which are easier and cheaper to produce and used in genome editing processes. Cleavage of staggered DNA, in contrast to the cleavage of blunt ends, might make forceful directional cloning a probability. Ultimately, the fact that the site of cleavage is situated far away from PAM might enhance homology-directed repair (HDR) *via* the second option for Cpf1 to probably re-cleave and re-initiate the process of HDR if it did not take place initially.

Type VI CRISPR is well-characterized by its signature protein C2c2, which targets single-stranded RNA phages. crRNA guides the C2c2 protein to initiate the cleavage, which takes place through two higher eukaryotes and prokaryotes nucleotide-binding (HEPN) domains once the effector complex faces the single-stranded RNA targets having protospacers, which match the sequence of crRNA.

The effector complex gets bound to the single-stranded RNA at the site of the target; however, the location of cleavage depends on the secondary structure of single-stranded RNA, with the site of cleavage occurring at exposed loop segments of single-stranded RNA. crRNA seed regions seem to be at the middle of crRNA and not at the 5′ or 3′ ends having double mismatches at the middle, thereby significantly decreasing the efficiency of the system. Fascinatingly, it seems as though the crRNA-effector complex and C2c2 get activated when they get successfully bound to the target single-stranded RNA; once they are activated, the effector complex is capable of cleaving other collateral single-stranded RNAs even when they are not particularly targeted.

## Delivery system of CRISPR-Cas

Currently, there are various types of delivery systems. For example, electroporation, nanoparticles, and hydrodynamics are used for the purpose of the CRISPR-Cas9 delivery system (Gori et al., 2015; Qin et al., 2015; Zuris et al., 2015). Non-viral vectors are preferred over the viral vectors though they have greater efficiency in delivering nucleic acids, as they are avirulent (Table 1).

**Table 1:** Various delivery tools for CRISPR-Cas process.

**Types of delivering system**	**Advantages and disadvantages**
Microinjection	It act as an efficient process of delivery but may induce cell damage and low throughput
iTOP	Acts as an efficient tool of delivery of Cas9 protein and sgRNA. But it does not show much of its efficiency in *in vivo* application.
Electroporation	It acts as an efficient tool in various CRISPR-Cas 9 delivery. But it has been observed to induce a significant amount of cell death as-well-as transfection
Hydrodynamic Injection	It acts as an efficient tool in various CRISPR-Cas 9 delivery. It does not prove to be suitable for large animals and medical purposes.
Lipid nanoparticles	It acts as an efficient tool in various CRISPR-Cas 9 delivery but possess low efficiency of action.
Polymer nanoparticles	Act as a delivery tool but possesses low efficiency of delivery

## Applications of CRISPRi in precision targeting of food bacterial biofilm genes

Since prokaryotes frequently do not possess robust pathways for repairing the endogenous DNA, double-strand cleavage *via* genomes of bacteria containing the mechanism of CRISPR-Cas is very threatening for the invasive foreign DNA. Even though this lethal characteristic poses many difficult challenges in the field of genome editing of bacteria, it also allows the utilization of the CRISPR-Cas system as an effective tool by acting as an anti-biofilm agent. Significantly, the systems of CRISPR-Cas are co-opted for enacting severe damage to specific populations of bacteria *via* sequence-specific self-targeting of the genome, resulting in the decrease of unwanted surrounding populations. If a strain possessing a CRISPR-Cas system is given a self-targeting spacer sequence, then CRISPR-Cas mediates the destruction of its own host genome. The severe impact observed following the targeting of the genomes of bacteria by CRISPR was first identified in 2013, in which it was demonstrated that self-targeting of the chromosome of the host resulted in an irreversible reduction (~10^5^) in the viable count (Vercoe et al., 2013).

As CRISPR acts as an anti-biofilm agent, it provides several novel solutions to challenges faced currently in the food industries, including fermentation industries specifically while managing mixed cultures. Mixed cultures are often utilized in fermentation industries for several reasons, such as for multiple step substrate transformation, reduction of the risk of developing infections by phages, and enhancing the yield of the product (Hesseltine, 1992). On the other hand, though mixed culture-mediated fermentation processes possess numerous benefits, there are several limitations as well. If the mixed culture is not characterized (such as an artisanal culture), it may become difficult to separate the strains, since many of the strains possess similar physiological characteristics. Moreover, contamination in the mixed culture due to external or foreign microbes may become challenging to regulate and remove the contaminants. Ultimately, it would be a daunting task to maintain the balance of ideal strains in the mixed culture, because the dynamics of populations are very much unique for particular combinations of microorganisms. Conventional strain isolation methods or regulation of the mixed-culture population or composition, including well-defined conditions for growth, antimicrobial peptides (AMPs), antibiotics, and bacteriophages, have hence provided us with just inadequate solutions.

### CRISPRi in food spoiling bacteria

Within the system of CRISPRi, a small guide RNA (gRNA) mediates the catalytic activation of the inactive dCas9 proteins to bind at or near the promoter region, thereby sterically hindering transcription initiation or elongation and leading to the silencing of gene expression. Systems of CRISPRi have been already proven to sterically inhibit gene transcription within model bacteria, such as *Bacillus subtilis* and *Escherichia coli* (Peters et al., 2016). Researchers have adopted the system of CRISPRi in *Pseudomonas fluorescens* (one of the major food spoiling bacteria) *via* the construction of a system containing two compatible plasmids (Noirot-Gros et al., 2018). One of the plasmids contains the gene dCas9, which is regulated by promoter P*tetA*, which can be induced by anhydrotetracycline presence (aTc) inside the medium of growth containing *Streptococcus pyogenes*. gRNA was constitutively expressed by gRNA (Table 2).

**Table 2:** Genes studied from food spoilage organisms responsible for food degradation.

**Food spoiling microbe**	**Genes involved**	**Mechanism of action**	**References**
Salmonella *enterica*	*Cas3*	*cas3* deletion upregulated the *lsrFGBE* genes in *lsr* (luxS regulated) operon related to QS and downregulated biofilm forming genes	Cui et al., 2020
*S. aureus*	*icaA*	CRISPRi plasmid vector, pBACi for *S. aureus* was designed to silence biofilm forming genes such as *icaA*	Sato'o et al., 2018
*Campylobacter jejuni*		CjCas9 was used for genome editing *in vivo*	Kim et al., 2017
*Listeria monocytogenes*		CRISPR RNA (RliB) is responsible for virulence of *L. monocytogenes* and binds to form RliB-CRISPR and is a substrate for polynucleotide phosphorylase (PNPase)	Espinoza-Mellado and Vilchis-Rangel, 2022

### Designing and assessment of CRISPRi

The function of the CRISPRi system was assessed in three strain isolates of *P. fluorescens*: Pf0-1, WH6, and SBW25. In each of these strains, the gene *mNG*, which encodes the mNeon-Green fluorescent protein, was subjected to regulation by constitutive Pc promoter and was integrated at identical chromosomal sites in all strains. Two pairs of gRNAs were designed; among them, one pair (Pc5 and Pc4) targets the initiation of transcription at the Pc promoter, while the second pair (Pc3 and Pc2) targets the elongation in transcription at a position, which overlaps the beginning of the open reading frame (ORF). gRNAs can target sites of DNA by either copying the non-template strand (Pc5, Pc2, and NT) or the template strand (Pc4, Pc3, and T). On inducing the expression of dCas9, the impact of gRNA on the intensity of fluorescence can be monitored at times by the use of flow cytometry. It was observed that targeting of the initiation of transcription by gRNAs Pc5 and Pc4 led to the highest reduction in mNG-mediated fluorescence in relation to control without gRNA (Noirot-Gros et al., 2018). On the other hand, a fraction of this reduction was found in the absence of an inducer, indicating that dCas9 is only expressed at some basal level (Noirot-Gros et al., 2018). The basal dCas9 expression had a minimum impact in the presence of Pc2 gRNA, which targets elongation in transcription and copies the NT strand (gRNA_NT_) in WH6 and SBW 25 strains. A full block of expression of *mNG* is expected to provide a 50% reduction in the intensity of fluorescence after each time the cell doubles due to the short time of maturation and high stability of protein *mNG* (Shaner et al., 2013). Under the assay conditions, generation time for WH6 and SBW25 strains was 120 min and 135 min, respectively. For both the strains, the identified reduction of fluorescence was slightly lower than that of the expected dilution of the protein *mNG* during cell division, thereby demonstrating a strong but incomplete silencing. Monitoring the impacts of targeting the T strand for the inhibition of elongation transcription by gRNA Pc3 showed a lowered reduction in fluorescence (3.2 times after 7 h) in comparison to the un-induced condition. This confirms the previous studies and their results regarding the significance of using the NT strand for maximum repression (Qi et al., 2013).

### CRISPRi gene silencing in morphogenesis and cytokinesis

For assessing the efficiency of the CRISPRi system, observable phenotypes are required to be generated in *P. fluorescens*, and for that, *mreB* and *ftsZ* genes need to be targeted because they are important for the survival of bacteria and on mutation show characteristic flaws in cellular shape or cell division.

On deleting the tubulin-like protein *ftsZ*, bacterial cells usually grow as non-septate and long filaments due to the failure in the assembly of a fully functional *ftsZ* division ring (Ortiz et al., 2016). The deletion of actin-like *mreB* cells of *E. coli* led to the loss of rod-shaped structure, giving rise to round enlarged cells (Ouzounov et al., 2016). Inside *B. subtilis*, which encodes numerous *mreB*-like proteins, each homolog depletion leads to aberrant morphology of the cellular phenotypes, including twisted, inflated, and wider cells, and finally leads to the lysis of the cells (Chastanet and Carballido-Lopez, 2012).

The proteins *mreB* (PFLU0863) and *ftsZ* (PFLU0952) within the SBW25 strain of *P. fluorescens* get silenced by the expression of gRNA that targets the *mreB* (*mreB*_*NT*_) and *ftsZ* (*ftsZ*_*NT*_) genes of the cells, which are being induced for the expression of dCas9. After incubating for 5 h at 25°C in the presence of an inducer at an aTc concentration of 100 ng/ml, the cells that express the *ftsZ*_*NT*_guide demonstrated a characteristic filamentation phenotype of the cell, which is mainly due to the knockdown of *ftsZ*_*NT*_. The morphological flaws appeared after 3–5 h of incubation, and this phenotype was even observed in cultures that were incubated overnight. Remarkably, these morphological defects can also be seen by using gRNA_T_, thereby targeting the *mreB* and *ftsZ* genes when the concentration of inducer was increased five times and incubated for longer periods. All these findings ensure the significance of using gRNA_NT_ for maximum repression and even indicate that slightly mild repression can be achieved with gRNA_T_. These findings suggest that CRISPRi is useful for silencing of genes in food biofilm-forming bacteria due to the phenotypic changes observed after long hours.

### CRISPRi-mediated mobility impairment and inhibition of formation of biofilm

The CRISPRi strategy is known to provide a fast silencing technique of genes, which is also stable over the due course of time. Since several phenotypes of bacteria associated with biofilm and mobility are usually measured after a period of 48 h, CRISPRi can also bring about gene silencing of the *gacS* (a two-component sensor kinase). Following induction in the presence of dCas9 expression, cells that express *gacS*_*NT*_ guide get fully impaired in their swarming ability after a period of 48 h for keeping with the tight motility regulation of *gacS* (Kim et al., 2014). The expression of *gacS*_*T*_guide also results in a remarkable defect in swarming. *gacS* silencing leads to stronger defects in the formation of biofilm in comparison to the strain lacking gRNA. Pellicles of biofilm formed at the liquid–air interface can be observed through confocal microscopy after staining the cells with a membrane-specific dye called FM1-43 biofilm tracer (green) or by staining the exopolysaccharides with Congo red dye (red). 3D structure of biofilms reveals that silencing of *gacS* resulted in the formation of a lesser cohesive and thinner pellicle with lower biomass of cells. Similarly, the matrix made up of exopolysaccharides showed unusual density and discontinuity, which means biofilm formation has been impaired due to the alteration in the physical characteristics of the pellicle. Hence, silencing of *gacS* mediated by CRISPRi entirely reproduces the phenotypic flaws in the formation of biofilm and surface motility, which are the hallmarks of biofilm-forming bacteria in food (Cheng et al., 2013). However, the strain with *gacS* knockdown seemed to be more resistant to exposure to hydrogen peroxide than the strain lacking it (Kim et al., 2014). Hence, it can be concluded that CRISPRi plays an important role in controlling the plate-based phenotypes of the bacterial cells that develop after 48 h of incubation and can be used as a control strategy for regulating the growth of food spoiling bacteria (Figure 2).

### CRISPRi-mediated silencing of c-diGMP signal pathway

CRISPRi-mediated silencing is considered to be popular and has an impact on the expression of genes at the level of the operon (Zhang et al., 2021). Hence, these operon structures can be assessed, since all the six genes of operon code for proteins that bind with c-diGMP. These genes comprise *alg44* (which encodes an alginate co-polymerase), *gcbA* (which codes for the DCG-promoting enzyme GcbA that helps in the formation of biofilm), and these two genes are the homologs of *bifA* and *dipA* genes, respectively, and code for enzymes having a characteristic phosphodiesterase (PDE) action in *Pseudomonas putida* and *Pseudomonas aeruginosa* (Zheng et al., 2018). Finally, the gene *rimA*, which encodes the single EAL domain protein called RimA, also exhibits a PDE action. These genes act at different phases of the formation of biofilm in *P. aeruginosa* (Valentini and Filloux, 2016). For examination of the operons, the gene profiling expression needs to be studied under various conditions of growth (Larsen et al., 2018). This profiling analysis indicated that *dipA* and *rimA* are the first two genes of the operon. Significantly, *rimA* downregulation is known to impact the downstream expression of genes, such as *rimK* and *rimB* (Little et al., 2016). In addition, the gene *bifA* is defined as a single unit of transcription; it is also a part of the operon and is thought to co-express with the downstream genes (Caspi et al., 2016). The gene *gbcA* seems to be a single cistron that is subjected to the conditions of growth during evaluation. On the other hand, its genomic assembly indicates that it can successfully be co-expressed with other downstream genes under the conditions of growth.

Ultimately, *alg44* is the third gene of the 12-gene cluster composing the *algD* operon. The *algD* operon is thought to be controlled by the alternate stress sigma factor σ 22 in *Pseudomonas*, and this factor gets repressed in the absence of biofilm-like conditions of growth (Wood and Ohman, 2012). The first six genes of the operon and their silencing appear to affect the neighboring gene transcription. For each of the genes, a gRNA_NT_ has been designed that targets the starting of the ORF, thereby creating a deletional mutant, except for the genes *rimA* and *gacS* because they have already been studied in much detail. Subsequently, the effects of gene silencing and deletion of gene phenotypes, such as the formation of biofilm and swarming motility, need to be measured.

### Effects of gene silencing on swarming motility

For comparison of the areas of swarming under similar conditions of growth for strains with CRISPRi-mediated deletion and silencing, all the deletional mutants were transformed with the pPFL plasmids expressing both gRNA and dCas9. The cells were grown on 0.4% agar plates in the presence of gentamycin (10 μg/ml), kanamycin (50 μg/ml), and aTc (100 ng/ml), and the swarming areas were measured at 25°C after a period of 48 h. CRISPRi-mediated silencing of *bifA, rimA*, and *dipA* remarkably decreased the ability of swarming, while *alg44* and *gcbA* silencing did not significantly impact the swarming ability.

The phenotypes of swarming that are obtained following gRNA_T_-mediated silencing are constant but are of decreased amplitude in relation to those found with gRNA_T_ in line with less effective silencing while targeting the template strand of DNA. Thus, this proves that the CRISPRi-associated strategy leads to the functional loss of phenotypes of motility.

### Gene silencing in c-DiGMP leads to loss of biofilm structure and formation

For assessing CRISPRi-based silencing of genes responsible for the formation of biofilm, EPS and bacterial cells present at the liquid-air pellicles are separately dyed and viewed through confocal microscopy. Biofilms formed by the cells lacking *RimABK* appear flakier and thinner with an irregular structure of the matrix formed by EPS (Little et al., 2016). Biofilm formed by cells lacking *dipA* look dense and very flat with a homogenous EPS partition. However, *BifA* depletion gives rise to a significant enhancement in the thickness of biofilms and clump formation in the matrix formed by EPS. Deleting the alginate co-polymerase *alg44* leads to the formation of a very thin biofilm having a more dispersed form of EPS matrix and synthesizes less amounts of alginate for the formation of exopolysaccharides (Whitney et al., 2015). Moreover, *GcbA* deletion slightly impairs the thickness of the biofilm-forming cells and gives rise to a modified EPS matrix.

**Figure 3:**
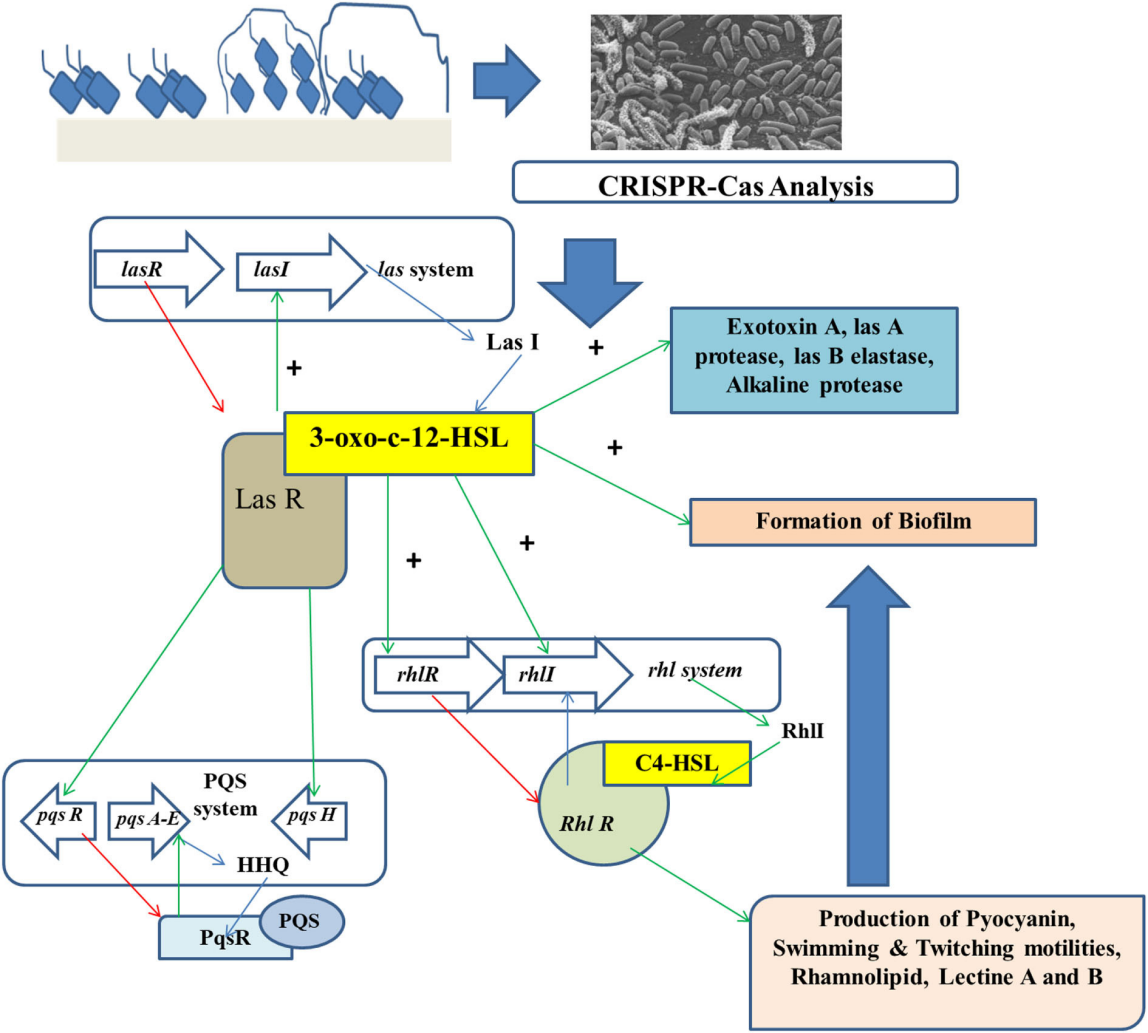
CRISPR-Cas analysis for preventing the growth of foodborne biofilm-producing organisms.

The 3D structures of biofilms are reconstructed for the quantitative analyses of biofilm roughness and thickness. CRISPRi-affected phenotypes are seen more in the deletional mutants than in the wild-type strains. In depleted and deletional conditions, a minor but statistically remarkable reduction in the thickness of biofilm can be observed for *alg44* and *gcbA* genes, while no remarkable change is found for *dipA* (Noirot-Gros et al., 2019). A more significant thickness can be found in depleted vs. deleted conditions for *bifA*, showing a statistically remarkable increase in mean (around 1.5 times), maximum thickness (about 2–3 times), and roughness (about 2.5 times). Such differences signify the incomplete silencing of genes (Peng et al., 2022). However, roughness gets slightly less significant in the case of deleted strains when compared to the depleted strains (Figure 3).

## Challenges in CRISPR-Cas technique

Wrong cutting of DNA is caused because of the off-target issues in the CRISPR-Cas systems, which may result in reducing the accuracy of the diagnosis and may also enhance the detection background and result in uncertainty. The innate side of CRISPR-Cas is its naturally occurring system, as it brings about an alteration in the sources and characteristics of the effector proteins and brings about differences in the structure and sequence of guide RNAs (Yin et al., 2021). The accuracy related to the off-target of CRISPR-Cas is a relative terminology, since with respect to the conventional technique like the PCR, it can be more accurate in detecting the targeted structure. Performing the CRISPR-Cas method after the process of normal PCR may facilitate double checking of the results and can be an accurate approach.

## Conclusion

The CRISPR-Cas system has mesmerized the scientific world right from the discovery of unusual repeat-spacer sequences in bacterial and archaeal genomes. The establishment of its function in the adaptive immune system in the year 2007 opened a new avenue for this field, resulting in the ultimate description of six different types of CRISPR-Cas systems, with each of them having its signature Cas proteins and typical interference mechanisms. The point of tipping took place over the last 4 years when the machinery of CRISPR-Cas was remodeled for editing of genomes. The food industry benefits much by adopting these techniques and tools for the synthesis of next-generation food cultures and for modulating and controlling mixed microbial populations. The prevalence of CRISPR-Cas in beneficial food microbes like probiotics and started cultures make fermentation processes a suitably positioned industry for many of the applications of CRISPR-Cas, including distinction and identification of almost related strains, protecting the important starter cultures against infection due to phages, vaccination of the strains preventing unnecessary uptake of plasmids, modulation or isolation of particular strains inside a mixed culture, and bacterial genome editing for creating industry-suitable bacterial work power. Moreover, CRISPR-Cas has been also found to be useful in working with detrimental microbes like spoilage microbes and pathogens, thereby making CRISPR-Cas an efficient tool for managing the population of microbes in the production processes of food. In addition to this, further research needs to be carried out for optimizing these applications and applying them to a broad spectrum of other industry-suitable microbes. The fact that remains to be observed is when the food industries will eagerly implement this technique of CRISPRi for the benefit of food science as a whole. Though this review has focussed on the applications of CRISPRi in protecting and manufacturing food, the application of this technique on a global scale is still not seen. With the pace and scale at which the CRISPRi system has developed newer strategies to prevent food spoilage, it is clearly evident that this can soon become a much-adopted technology that can be used for further research and development along with maximizing the quality of food products and safety during their manufacturing processes.

## Author contributions

Conceptualization: DL, MN, SG, TS, and RR. Methodology: DL, MN, TS, SP, SG, and MM. Formal analysis: DL, MN, MM, and RR. Investigation: DL, MN, and RR. Original draft preparation: DL, MN, HE, SP, TS, MK, and RR. Reviewing and editing: DL, MN, SP, TS, MK, and RR. All authors have read and agreed to the published version of the manuscript.

## Conflict of interest

Author SP was employed by the company NatNov Bioscience Private Limited, Balasore, India. The remaining authors declare that the research was conducted in the absence of any commercial or financial relationships that could be constructed as a potential conflict of interest.

## Publisher's note

All claims expressed in this article are solely those of the authors and do not necessarily represent those of their affiliated organizations, or those of the publisher, the editors and the reviewers. Any product that may be evaluated in this article, or claim that may be made by its manufacturer, is not guaranteed or endorsed by the publisher.
